# Control of Urban Zika Vectors: Should We Return to the Successful PAHO/WHO Strategy?

**DOI:** 10.1371/journal.pntd.0004769

**Published:** 2016-06-01

**Authors:** Paul Reiter

**Affiliations:** Institut Pasteur, Paris, France; University of California, Davis, UNITED STATES

We keep hearing that vector control is our only weapon against Zika.

The problem is: How?

We assume that the urban vectors of the virus are *Aedes aegypti* and *Ae*. *albopictus*. They are also the principal vectors of urban dengue and chikungunya, in which case the spectacular increase in the global prevalence and incidence of both diseases [1] is harsh condemnation of our current control strategies. The stark truth is that over the past 50 years no country anywhere in the world (with the possible exception of Singapore) can claim sustained suppression of transmission of these viruses [2].

A plethora of factors has been blamed: explosive growth of urban areas, globalization of pathogens and vectors, limited government resources, excessive reliance on insecticides, ineffectual application technology, incorrect application methods, insecticide resistance, poor training of field personnel, the “throw-away society”, a “quick fix” mentality, inadequate garbage collection, irregular water supply, inadequate public education and overemphasis on the “top down” role of governments rather than the “bottom up” role of the community, and more.

In the past, nevertheless, there were two remarkable examples of success: the source-reduction campaigns that began at the turn of the 20^th^ century, and the *Ae*. *aegypti* Eradication Campaign—coordinated by the Pan-American Health Organization (PAHO)—that followed in the late 1940s. The goal of the latter was complete eradication of the species from the entire western hemisphere and indeed, by 1962, eighteen countries had been declared totally free of the mosquito and of dengue [3]. Unfortunately, for a variety of reasons—including insecticide resistance and failure to sustain efforts in regions where the campaign had been successful—the project was abandoned and both mosquito and virus quickly regained their lost territory.

The challenges that confronted the eradication campaign are dwarfed by the obstacles that we face today. The problem is primarily urban: *Ae*. *aegypti* is ubiquitous and abundant in urban areas throughout the tropics. In addition, in the past 30 years, *Ae*. *albopictus* has invaded many tropical and temperate regions world-wide.

**Fig 1:**
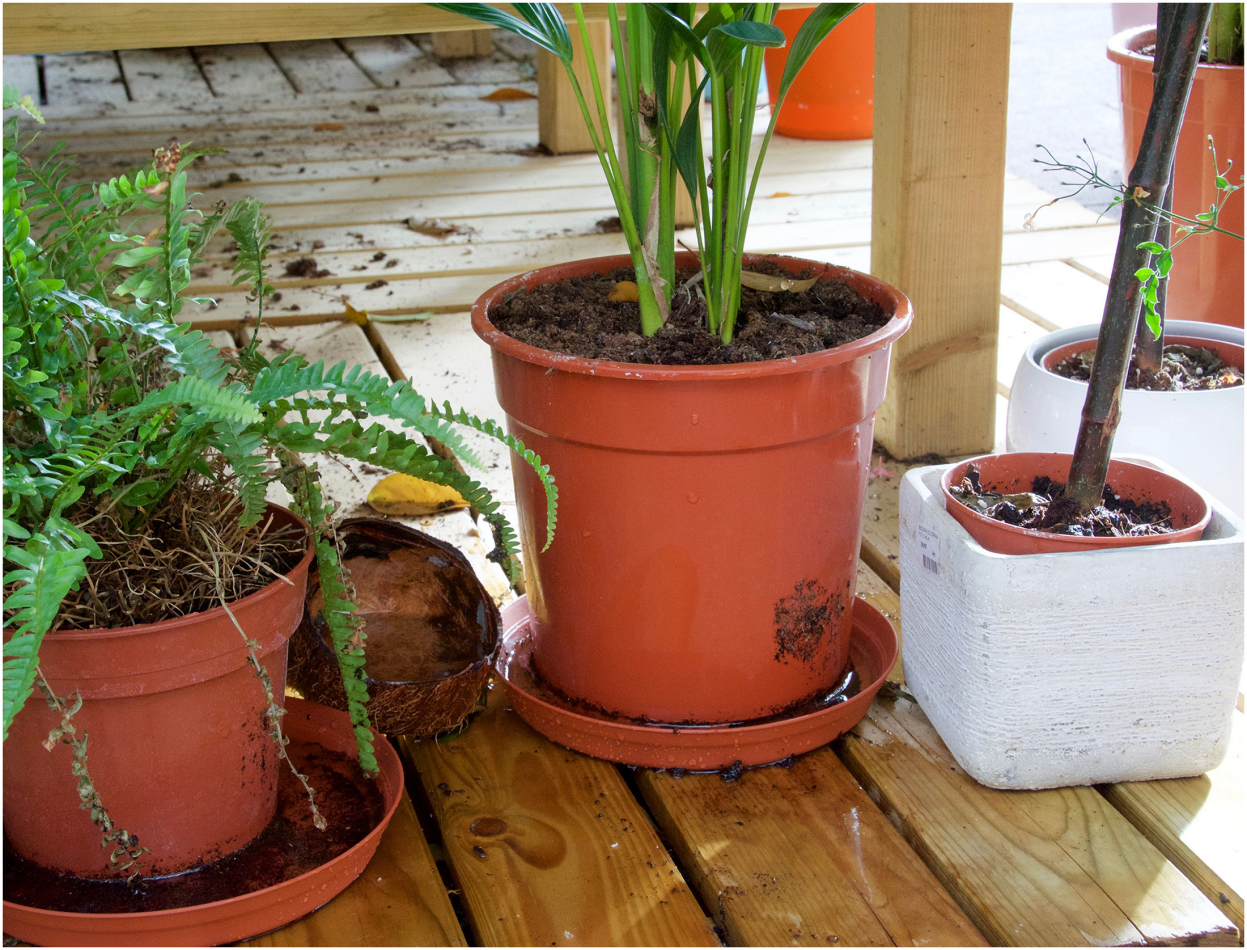
*Aedes aegypti* and *Ae*. *albopictus* are forest species that breed in tree-holes, plant axils, fruit husks and other small natural containers. They thrive in the urban environment by exploiting the abundance of artificial containers. Many such containers are hard to detect but “skip ovipositon” may ensure that residual treatments are effective even if not all sites are detected/treated.

To understand the abundance of both we must be mindful of their origins as forest species. In that habitat they did not breed in ground-pools or marshlands but in a well-defined niche: tree-holes, plant axils, fruit husks, rock-holes and other small natural containers. They have adopted the human peri-domestic environment by exploiting the profusion of artificial containers in the human jungle—water storage vessels, discarded tires, blocked gutters, broken china, cracked buckets, defunct toilet bowls, saucers under flowerpots, flower vases, water-storage vessels, abandoned kiddie play-pens and so-on (Fig 1).

In the period 1950–2014 the world’s urban population rose from 746 million to 3.9 billion. Much of this growth was in the tropics; 80% of the populations of Latin America and the Caribbean, for example, now live in urban areas [4] and in most of the region they share their habitat with one or both *Aedes* species. Compared to their forest ancestors, these mosquitoes live in a burgeoning paradise, and the obstacles to significant control, even on a small scale, would appear insurmountable.

I believe that the spectacular success of the PAHO campaign can be attributed to a single aspect of the behavior of the mosquitoes: female *Ae*. *aegypti* do not lay “all their eggs in one basket”. Instead, they “skip oviposit”—small numbers of eggs (often only a single egg) at many sites [5]. In the field they will lay 60–80 eggs per gonotrophic cycle and thus must visit many sites. This behavior may be a strategy to avoid overcrowding in a niche where larval nutrition is limited, and/or to minimize risks inherent in temporary sites.

In the eradication campaign, the principal approach was “perifocal” treatment: field operators searched for infested containers and sprayed them plus surrounding surfaces to a diameter of about one meter with DDT [3]. Residual treatments of this kind kill mosquitoes by contact.

Infested containers are remarkably hard to detect, even by the most diligent persons. Faced with a dreary routine of search-and-find, field-workers would inevitably have missed many sites, particularly those not in the usual categories (top on my list of such “specials” include the baptismal font in a church and the insides of a metal sculpture). Thus it is inconceivable that every container in all those countries was located and treated. On the other hand, with “skip oviposition”, even if only a portion of infested sites was treated, there would have been a high probability that females would encounter a treated site; they were engaged in Russian Roulette with multiple bullets.

In the current Zika pandemic, the media repeatedly show “fogging machines” mounted on road vehicles and/or troops of workers on the ground “space-spraying” residential areas with insecticidal aerosols. The impact of such treatments is ephemeral at best because they are mainly effective while the droplets remain airborne, a matter of minutes. By contrast, peri-focal treatments with a residual insecticide could create numerous lethal sites that remain active for several months and actually lure the egg-laying female.

I believe we should consider a return to peri-focal treatments. DDT is not an option (although it is now re-approved by the World Health Organisation (WHO) for mosquito control in certain circumstances) but novel formulations of other insecticides are available that may serve the same purpose. In one such product, tiny crystals of deltamethrin are embedded in a patented liquid polymer that dries to a stable, rain- and sun-proof lattice. According to the manufacturers, treatments remain effective by slow release of the insecticide for at least three months. Field trials of indoor residual spray (treatment of the walls inside houses) have demonstrated good kill by this formulation but the approach is far more intrusive than focal treatments outdoors.

Field trials to assess the viability of perifocal treatments could be completed in a matter of months. If results are favorable, the insecticide could be applied with widely-available hand-carried sprayers. Training requirements would be minimal and operators would not have to don the full-body suits used with hand-foggers; these are understandably disquieting to the public.

I am not invoking a panacea for *Aedes*-borne urban disease but I suggest that a well-managed treatment-campaign, bolstered by community-based source reduction and other strategies, might well be as successful as was the PAHO campaign. In areas too large or too difficult to treat, triage to select potential hot-spots of transmission—schools, hospitals, prisons, markets, outdoor cafés and restaurants and so on—might at least help emergency services to cope by flattening the epidemic curve.

The curse of such campaigns is, of course, sustainability—it is hard to justify funding for a problem that no longer exists (although no-one questions the need for sustained garbage collection and other community services). In addition, long-term use of insecticides has several drawbacks, particularly the evolution of resistance. Happily, a number of exciting new approaches are in the pipeline—transgenics with a dominant lethal gene [6], the release of strains infected with *Wolbachia* bacteria [7], and auto-dissemination of juvenile hormone mimics [8]—although all are some way from mass application.

Ultimately, vaccination may be the preferred weapon, particularly if combined with vector control [9], but vaccine development and approval takes time, generally a matter of years. The lesson of Zika is that other viruses may be waiting in the wings for which the same delays will apply.

For this reason, the need for effective vector control will continue.
